# Interaction Mechanism of Thermal and Mechanical Field in KDP Fly-Cutting Process

**DOI:** 10.3390/mi12080855

**Published:** 2021-07-21

**Authors:** Chenhui An, Ke Feng, Wei Wang, Qiao Xu, Xiangyang Lei, Jianfeng Zhang, Xuelian Yao, Haibo Li

**Affiliations:** 1 Laser Fusion Research Center, CAEP, Mianyang 621900, China; xuqiao@vip.sina.com (Q.X.); leixiangyang2@163.com (X.L.); xxxxxx5726@vip.sina.com (J.Z.); xlyao93@163.com (X.Y.); haiya126@sina.com (H.L.); 2 School of Mechanical and Electrical Engineering, University of Electronic Science and Technology, Chengdu 611731, China; duying0108@163.com (K.F.); wangwhit@163.com (W.W.)

**Keywords:** potassium dihydrogen phosphate (KDP) crystal, single point diamond fly-cutting, interaction mechanism, chip morphology, phase transition temperature

## Abstract

As an important nonlinear optical material, potassium dihydrogen phosphate (KDP) crystal is used in high-power laser beams as the core element of inertial confinement fusion. It is the most general method of single point diamond fly-cutting (SPDF) to produce high precision and crack-free KDP surfaces. Nevertheless, the cutting mechanism of such material remains unclear, and therefore needs further analysis. Firstly, the stress field, cutting force and cutting temperature under different working conditions are calculated by a KDP crystal cutting simulation model. Then, the rules and the cause of change and interaction mechanisms of force and temperature are analyzed by comparing the measurement experiments with simulations. Furthermore, the causes of chip formation and micro-cracks on the machined surface are analyzed based on thermo-mechanical coupling and chip morphology. The conclusion can be deduced: Although the temperature has not reached the phase transition temperature during the finishing process, under high cutting speeds and large unformed chip thickness, such as semi-finishing and roughing, the temperature can reach up to 180 °C or higher, and KDP crystals are very likely to phase transition—chip morphology also verifies this phenomenon.

## 1. Introduction

Potassium dihydrogen phosphate (KH_2_PO_4_, KDP) is a unique artificial crystal that can grow to a diameter greater than 500 mm, and it has excellent nonlinear optical properties [1]; therefore, it is the only nonlinear optical element material that can be used in high-energy laser beams [2]. However, due to its disadvantageous properties such as deliquescence, fragmenting and strong anisotropy, it is less possible to process the KDP crystal by grinding, polishing and other traditional methods [3,4]. Hence, single point diamond fly-cutting is the most feasible process method for KDP crystal, and because of the high requirement of the crystal elements such as nanoscale roughness and micron level surface accuracy, the ultra-precision fly-cutting machine tools are adapted and the fine finishing process is analyzed [5]. A single point diamond fly-cutting machine with a large diameter cutting head, which can effectively avoid the mentioned disadvantages and reduce the effect of the material anisotropy influence, is the most common machining method for large caliber KDP crystal components [6].

In the KDP crystal fly-cutting process, the cutting speed could reach up to 10 m/s, which is higher than that in ordinary turning processes. Under such a high cutting speed, on one hand, the crystal material near the cutting zone will incur a large plastic deformation, and on the other hand, it causes severe friction between cutting tools and workpieces. The plastic deformation and severe friction would produce heat, which would increase the material’s temperature near the cutting zone [7,8], resulting in some surface defects such as phase transition, recrystallization and micro-cracks, especially for the KDP crystal of which the phase transition temperature is only 182.3 °C [9].

Lo [10] established a finite element model to represent the cutting process by using diamond tools with different rake angles, taking the influence of the edge radius into account. Zong [11,12] established a finite element model to simulate the entire diamond turning process, and found an optimal selection of tool geometries for the KDP cutting process, i.e., −25° rake angle and 8° clearance angle [13]. A new constitutive model was used by Wang [14] to simulate the cutting process of KDP crystal, which combines the anisotropic elastic and pressure-dependent plastic model and is performed to investigate the influence of cutting parameters on the brittle ductile transition depth and cutting force. In [15], the authors took research on the thermal field of DKDP crystal cutting and explored the influence of process parameters on the cutting temperature. However, there are few studies on the thermo-mechanical coupling phenomenon in the KDP crystal cutting process.

At the same time, there are many scholars involved in the research on material properties. Zhang [16] tested the mechanical parameters of KDP crystals and found that brittle failure is easier to appear in [100] crystal orientation than [001] crystal orientation, and the compressive strength is much higher than tensile strength. In 2015, Ding [9] studied the high temperature thermal behavior and thermal dehydration reaction of KDP crystal, and the main conclusion is that the KDP crystal is still tetragonal and has no phase transition of the monoclinic phase at 183 °C, and then the dehydration reaction begins to decompose at 207 °C. Hou [17] used XRD (X-ray diffraction) to analyze the residual stress of the subsurface of KDP crystal. It is found that the residual stress in the vertical [112] plane is higher than other planes at different cutting depths, and its value is about 30 MPa, which is about 7 times that of the parallel [112] plane residual stress. In 2018, Huang [18] studied the KDP crystal cracking caused by temperature inhomogeneity, and found that when the crystal has an internal temperature difference of 4 °C in a small spatial scale of about tens of millimeters, the maximum residual heat stress would be very close to or even exceed the tensile strength of 6.67 MPa [19], which made the crystals easier to crack.

There are many kinds of methods that can be used to measure the online temperature in the machining process, and the most common method is using embedded thermocouple probes [16]. Thus, this kind of probe must be embedded in the measured position, and they need a high coefficient of heat conduction of the measured objects, both of which cannot be fulfilled in the KDP cutting process [20,21]. In the KDP crystal fly-cutting process, the linear velocity of the diamond tool is above 10 m/s, and measuring the temperature distribution in the KDP cutting process is extremely difficult due to the small shear area and low heat production [21]. Therefore, a kind of high-speed infrared camera is necessary in this situation which can meet both requirements of remote measurement and high temporal resolution. The ImageIR^®^ 5300 has a sampling frequency as high as 12 kHz, and its maximum resolution is 0.02 °C, which is the ideal equipment for this experiment.

In short, although lots of research works are published about the cutting force and the temperature change in the KDP crystal fly-cutting process, few studies have combined the temperature and stress analysis together, although both of which would have effects on the material removal mechanism. This paper is based on the anisotropic finite element model of KDP crystal to study the interaction of the thermal and mechanical field in KDP crystal cutting and its influence on chip morphology. It is the main method by combining simulations on the stress field, temperature field and cutting force with experimental verification results. Studying the interaction of the thermal and mechanical field is helpful to understand the material removal mechanism, verify the material changes in the cutting process, and reveal the phase transformation and surface defects that may be caused by the heating produced in the fly-cutting process.

## 2. Simulation

### 2.1. Simulation Modeling

**Figure 1:**
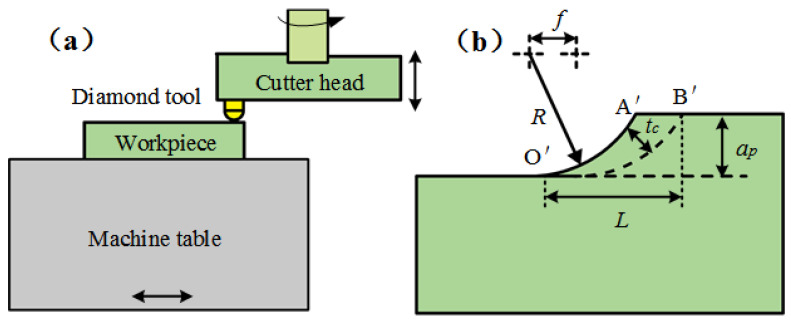
(**a**) Fly-cutting schematic diagram; (**b**) Undeformed chip thickness.

As is shown in Figure 1a, in the KDP fly-cutting process, the workpiece is placed horizontally and fed at a constant velocity, while the spindle with the diamond cutting tool rotates at a given speed. Along the main cutting direction, the section of the removed workpiece material produced by the arc edge tool is circular, which can be regarded as the superposition of many sections with different cutting depths, so the three-dimensional problem can be transformed into a series of two-dimensional plane problems with certain thicknesses. In Figure 1b, the area enclosed by the three points of $O^{\prime }A^{\prime }B^{\prime }$ is an undeformed chip.

(1) $$t_{c}=R-\sqrt{R^{2}+f^{2}-2f\sqrt{2Ra_{p}-a_{p}{}^{2}}}$$

The max value of the unformed chip thickness *t_c_* can be calculated according to Equation (1), where *R* is the nose radius and the value is 5 mm, *f* is feed and the value is 10 μm/rev according to the actual situation, and *a_p_* is the cutting depth.

**Figure 2:**
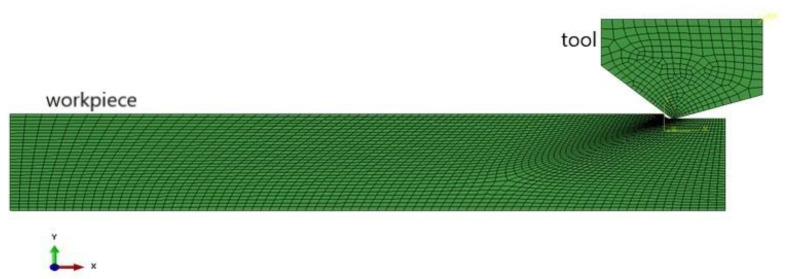
KDP crystal cutting process finite element simulation model.

Figure 2 shows the finite element simulation model of KDP crystal. The KDP crystal workpiece is rectangular with a size of 100 μm × 30 μm. The 3600 four-node plane strain units (CPE4) are used to complete the mesh. The bottom of the KDP crystal is completely fixed in a horizontal manner. Compared with the soft KDP crystal, the diamond tool is far harder and is often regarded as a rigid body and moves to the left of the workpiece at a certain speed. The parameters of the diamond tool are as follows: the edge radius is 120 nm, the tool clearance angle is 18°, and the tool rake angle is −45°. The 300 four-node plane strain units are used to mesh the diamond tool.

### 2.2. Material Properties

#### 2.2.1. Diamond

The KDP crystal fly-cutting process mainly involves two different materials. The processed material is KDP crystal, which is characterized by low hardness and high brittleness, and the tool material is natural diamond, which is the hardest material in the world. The hardness of diamond is more than 10 times that of KDP crystal. The specific material properties of natural diamond are shown in Table 1.

#### 2.2.2. KDP Crystal

Since the KDP crystal is an anisotropic material, the structure of the KDP crystal is a tetragonal system, and the matrix of the elastic phase is composed of six mutually independent parameters to characterize the stress–strain relationship of the KDP crystal. The stress–strain relationship inside the KDP crystal material can be expressed by the stiffness matrix of the material, as shown in Equation (2):

(2) $$\left[\begin{array}{c} \sigma_{x}\\ \sigma_{y}\\ \sigma_{z}\\ \tau_{xy}\\ 0\\ 0 \end{array}\right]=\left[\begin{array}{cccccc} C_{11} & C_{12} & C_{12} & & & \\ C_{12} & C_{11} & C_{13} & & & \\ C_{13} & C_{13} & C_{33} & & & \\ & & & C_{44} & & \\ & & & & C_{66} & \\ & & & & & C_{66} \end{array}\right]\left[\begin{array}{c} \varepsilon_{x}^{}\\ \varepsilon_{y}\\ 0\\ \gamma_{xy}\\ 0\\ 0 \end{array}\right]$$

There, *ε_x_, ε_y_,* and *γ_xy_* are the strain components, and *C*_11_*~C*_66_ are the elastic constants of KDP crystal.

Table 2 shows the KDP crystal stiffness coefficient values, which can express the properties inside the KDP crystal.

When the KDP material is in the plastic stage, the final model of the material constitutive equation can be derived from Equation (3):

(3) $$\left\{ \begin{array}{l} \sigma =471.227\varepsilon^{0.1721}(\sigma \geq 181.725MPa)\\ \sigma_{ture}=\sigma (1+\varepsilon )\\ \varepsilon_{p}=\ln(1+\varepsilon )-\sigma_{ture}/E \end{array}\right.$$

where $\sigma_{ture}$ is the true rheological stress and $\varepsilon_{p}$ is plastic deformation [10].

Temperature is accompanied by the cutting process, so temperature analysis is an integral part of the finite element simulation of KDP crystal fly-cutting. The heat transfer in the shear zone would change the plastic deformation; therefore, the surface micro structure may be changed. The diamond tool is a rigid body relative to the KDP crystal and only heat transfer is calculated, and the KDP crystal workpiece is a deformable body, and the thermal and mechanical effects are analyzed in the simulation. In the heat transfer analysis of the KDP crystal, the heat conduction and convection calculations are performed. Relative thermal parameters of diamond tools and Type II KDP crystals, for example, the specific heat capacity C, thermal conductivity α, thermal expansion coefficient *λ* and thermal emissivity *k*, are listed in Table 3.

**Figure 3:**
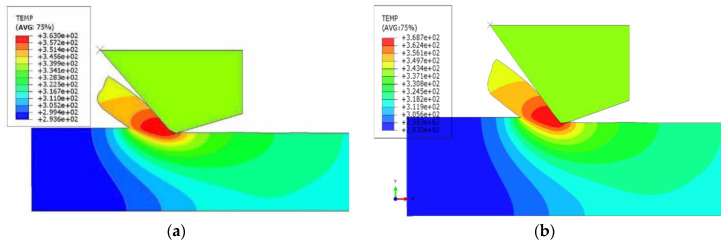
Temperature distribution along different crystal orientations: (**a**) [100] crystal orientation, (**b**) [001] crystal orientation.

Figure 3 shows the temperature in different directions—the cutting depth is 2 μm (the max value of the unformed chip thickness is 297 nm) and the line velocity of the diamond tool is 10 m/s. (a) The cutting direction is [100] crystal orientation, and the temperature is 90 °C. (b) The cutting direction is [001] crystal orientation, and the temperature is 96 °C, indicating that the anisotropy has a certain influence on the cutting temperature of the KDP crystal, and it is better to cut along [100] crystal orientation as a lower hit is being produced in this direction.

## 3. Experiment

### 3.1. Cutting Experiment

**Figure 4:**
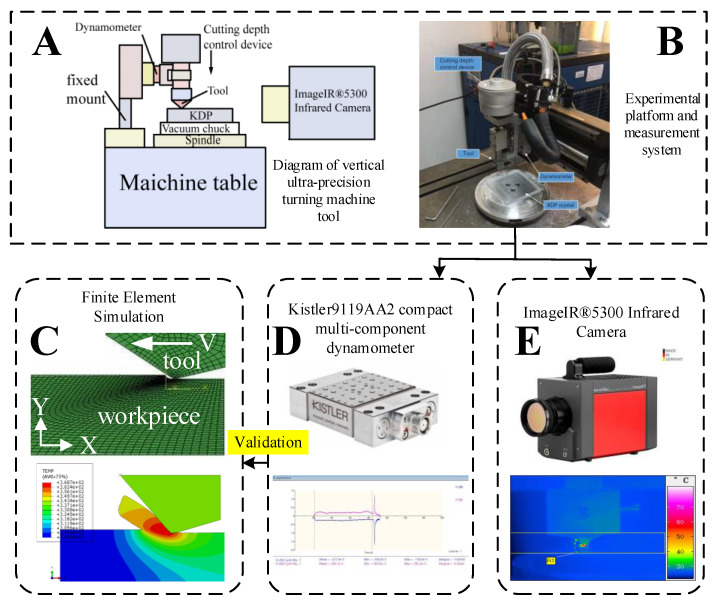
Experimental platform and measurement system: (**A**) Diagram of vertical ultra-precision turning machine tool; (**B**) Experimental platform and measurement system; (**C**) Finite Element Simulation; (**D**) Kistler9119AA2 compact multi-component dynamometer; (**E**) ImageIR^®^5300 Infrared Camera.

As shown in Figure 4, in order to measure the force and temperature in the process of KDP crystal, a vertical ultra-precision turning machine tool was used—the spindle rotation error is 0.1 μm, the feed repetition accuracy is 0.1 μm, and the spindle speed can reach 8000 r/min.

The tool selected for the ultra-precision cutting machine tool was a diamond arc-edge tool. The parameters of the tool are consistent with those in the simulation model, as shown in Table 4.

The unformed chip thickness and cutting speed both need to be analyzed in order to obtain the effect of single process parameters on KDP crystal fly-cutting, and orthogonal experiments of cutting speed and unformed chip thickness are used to complete the influence of single parameters on the cutting force and cutting temperature. The experimental parameters were designed as shown in Table 5.

In the KDP crystal fly-cutting process, the process parameters are small, such as the μm range cutting depth, and the cutting forces are always less than 0.1 N; therefore, a high accuracy measurement device for the cutting force is required. Figure 4D shows the Kistler9119AA2 compact multi-component dynamometer used in this experiment. The dynamometer is compact in design, has high sensitivity and natural frequency, and has small temperature error. It is very suitable for measuring the change in cutting force in ultra-precision machining processes.

In the KDP crystal fly-cutting process, the diamond tool rotates with the spindle at a high speed, the line velocity of the cutter can exceed 10 m/s, and the contact period between the tool and KDP crystal is very short, so it is necessary to use a temperature measurement system with a high frequency response. The IR^®^5300 infrared camera used in this experiment is shown in Figure 4E. Its sampling frequency is as high as 12 kHz, its maximum resolution is 0.02 °C, and it can measure the weak temperature changes in high-speed cutting areas.

### 3.2. KDP Chip Morphology Observation Experiment

Studying the chip morphology formed by high-speed cutting is helpful to explain the chip formation mechanism. The chip forming process is the plastic deformation of the workpiece material sliding along the shear surface after being pushed by the tool rake face. Chip shape can explain the deformation mechanism and process in the cutting process, and indirectly explain the quality of the workpiece surface. Based on the observation of chip morphology, the influence of process parameters and temperature on chip formation can be analyzed by combining the simulation and experimental results.

In this experiment, the NeoScope Jcm-5000 desktop scanning electron microscope was used to observe the KDP chip morphology. The surface morphology of the chips after finishing, semi-finishing and roughing was observed, respectively, and the process parameters are consistent with those in the simulation and measurement experiments.

## 4. Results

### 4.1. Results Comparison of Cutting Force 

**Figure 5:**
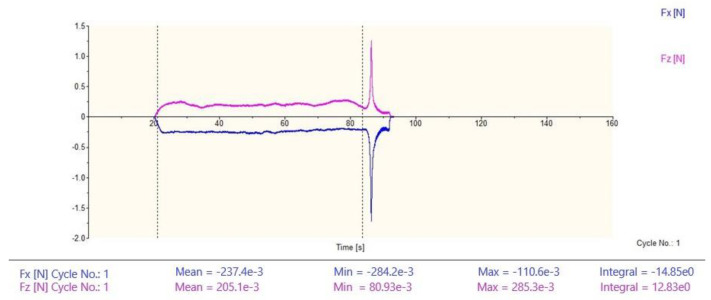
Cutting force measurement chart.

Figure 5 shows the cutting force measurement chart at the cutting speed of 3 m/s and the unformed chip thickness of 429 nm. F_x_ is the horizontal cutting force and F_z_ is the vertical cutting force. The data between the two dotted lines in the figure are the cutting force measurement results from the cutting tool to the workpiece in the cutting process. From the figure, we can calculate the average values of the horizontal and vertical cutting forces, which are 0.237 N and 0.205 N, respectively.

**Figure 6:**
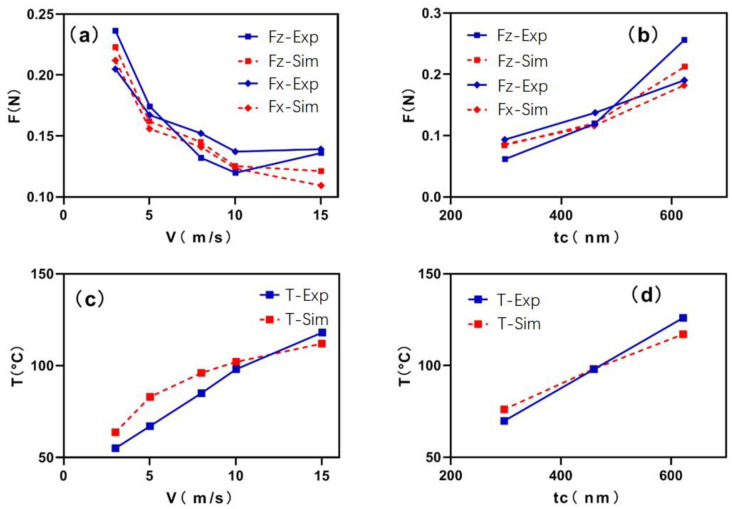
Variation of cutting force and temperature under different cutting conditions. (**a**) Comparison of cutting forces at different cutting speeds under the same unformed chip thickness of 429 nm. (**b**) The comparison of cutting forces at different unformed chip thicknesses under the cutting speed of 10 m/s. (**c**) Comparison of cutting temperatures at different cutting speeds and unformed chip thickness of 429 nm. (**d**) Comparison of cutting temperature at different cutting and depth cutting speed of 10 m/s.

Figure 6a shows the comparison of cutting forces at different cutting speeds under the max unformed chip thickness of 429 nm. In the figure, it can be found that: (1) cutting speed ranged from 3 m/s to 10 m/s, both F_Z_ and F_x_ decreased by 20–30% with the increase in cutting speed, meanwhile the simulation results and experimental results have the same trend and the numerical values are close, which shows that the simulation is in line with the actual situation. (2) When the cutting speed increased from 10 m/s to 15 m/s, the variation trends of the measured and simulated cutting force were different. The simulated cutting force F-S_im_ decreased with the increase in the cutting speed, but the experimental cutting force F-E_xp_ increased. The main reason is that the chip generation rate is higher than the chip discharge rate, and the cutting force is affected by the chip accumulation in the actual process; in addition, with the increase in machine speed, the stability of the machine tool will become worse, which affects the measurement of cutting force.

Figure 6b shows the comparison of cutting forces at different unformed chip thicknesses under the cutting speed of 10 m/s. In the figure, F_z_ and F_x_ increased with the increase in depth. When the cutting speed remains constant, the measured and simulated values of cutting force at different cutting depths are basically the same trend, and the numerical values are close, which shows that the finite element simulation model is in line with the actual situation and therefore can be convinced.

### 4.2. Results Comparison of Cutting Temperature 

**Figure 7:**
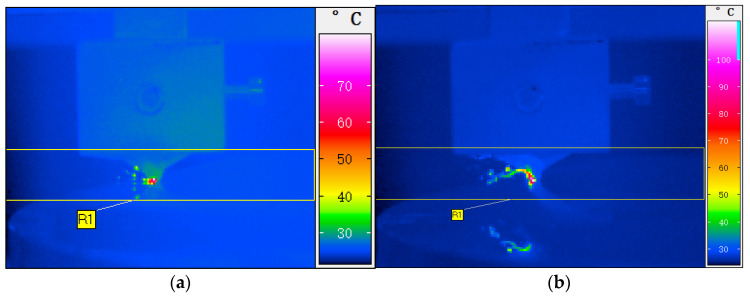
Cutting temperature images: (**a**) Cutting speed 5 m/s, (**b**) Cutting speed 10 m/s.

Figure 7a,b show the cutting temperature images of KDP crystal taken by the ImageIR^®^300 infrared thermal camera. When the cutting speeds are 5 m/s and 10 m/s, respectively, the unformed chip thickness remains 429 nm. The maximum values of the measured temperatures are 70 °C and 98 °C, respectively.

Figure 6c shows the comparison of cutting temperature results at different cutting speeds under the unformed chip thickness of 429 nm. As shown in the figure, firstly, as cutting speed increased, the cutting temperature increased by 260%. However, when the cutting speed increased to a certain extent, the growth rate of cutting temperature slowed down and the influence of cutting speed weakened gradually. Secondly, the maximum difference between the simulation temperature and the experimental results is less than 10 °C, which shows that the accuracy of the finite element simulation model can be accepted. Thirdly, when the unformed chip thickness was 429 nm and the cutting speed was 15 m/s, the simulation temperature of the chip reached 112 °C and the actual temperature reached 118 °C. Under such a high temperature and high speed deformation, the internal structure of the KDP crystal may change with the observation in Section 4.5, so high-speed cutting such as 15 m/s should be avoided as much as possible in the actual process.

Figure 6d shows the comparison of cutting temperature results at different unformed chip thicknesses under the cutting speed of 10 m/s. As shown in the figure, with the increase in unformed chip thickness, the cutting temperature increased by 74–90%, and the trend of chip temperature change is basically the same. Then, the simulation temperature of chips is close to the experimental results, and the maximum difference is less than 10 °C, which also validates the accuracy of the simulation model.

### 4.3. Influence of Cutting Speed

**Figure 8:**
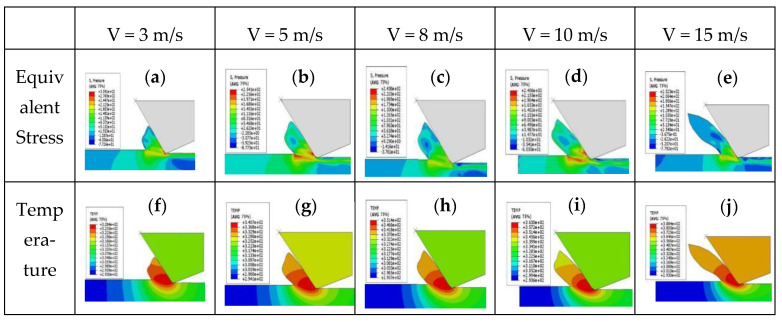
Stress distribution and temperature distribution of KDP crystal at different cutting speeds with unformed chip thickness of 429 nm: (**a**) Equivalent Stress of V = 3 m/s; (**b**) Equivalent Stress of V = 5 m/s; (**c**) Equivalent Stress of V = 8 m/s; (**d**) Equivalent Stress of V = 10 m/s; (**e**) Equivalent Stress of V = 15 m/s; (**f**) Temperature of V = 3 m/s; (**g**) Temperature of V = 5 m/s; (**h**) Temperature of V = 8 m/s; (**i**) Temperature of V = 10 m/s; (**j**) Temperature of V = 15 m/s.

Figure 8a–e show the stress distribution at an unformed chip thickness of 429 nm (the cutting depth is 5 μm) and cutting speeds of 3 m/s, 5 m/s, 8 m/s, 10 m/s and 15 m/s. The compressive stress distribution is mainly found in the chip, and the maximum compressive stress occurs near the boundary between the workpiece and the chip along the shear direction, and the values are 309 MPa, 254 MPa, 243 MPa, 240 MPa, and 232 MPa, respectively. The maximum cutting stress at 15 m/s decreases by nearly 24% compared with that at 3 m/s.

Figure 8f,j show the temperature distribution of KDP crystals with an unformed chip thickness of 429 nm and different cutting speeds. The cutting speeds are 3 m/s, 5 m/s, 8 m/s, 10 m/s, and 15 m/s, respectively. The maximum temperatures of the chips in the figure are 55 °C, 67 °C, 85 °C, 98 °C, and 112 °C, respectively.

In Figure 8, when the unformed chip thickness was 429 nm, as the cutting speed increased: (1) The cutting force at 15 m/s decreased by nearly 25% compared with that at 3 m/s, and the maximum cutting stress decreased about 20% from 3 m/s to 15 m/s. (2) Cutting temperature increased from 58 °C to 112 °C, which increased by 93%. (3) In the process, the cutting force decreased with the higher cutting speed, and the most likely explanation can be deduced: Most chips contacted with the rake face, and with cutting speed increasing, the temperature gradually increased and the material softened, then the workpiece material was easier to remove and the friction between chip and tool decreased. So as cutting continued, the material softening level increased and the cutting force tended to be stable gradually, which was related to cutting temperature, so that the growth rate of temperature decreased and the cutting temperature became stable gradually. (4) After KDP crystal processing, there is tensile stress on the subsurface, which is about 3 MPa~10 MPa. (5) There is also a residual temperature of 30 °C~50 °C on the machined surfaces. 

### 4.4. Influence of Unformed Chip Thickness

**Figure 9:**
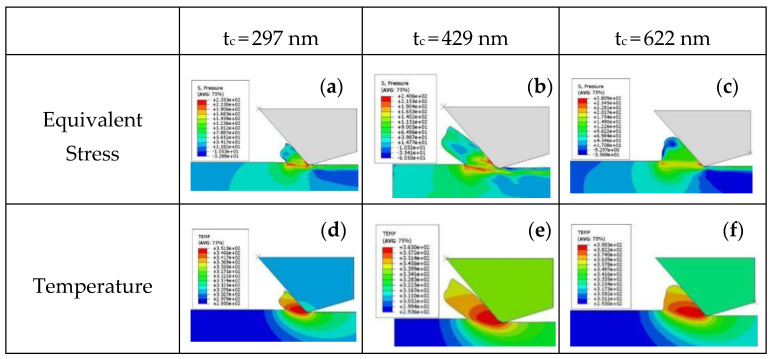
Stress distribution and temperature distribution at different unformed chip thicknesses at cutting speed of 10 m/s: (**a**) Equivalent Stress of tc = 297 nm; (**b**) Equivalent Stress of tc = 429 nm; (**c**) Equivalent Stress of tc = 622 nm; (**d**) Temperature of tc = 297 nm; (**e**) Temperature of tc = 429 nm; (**f**) Temperature of tc = 622 nm.

Figure 9a–c show a stress distribution with unformed chip thicknesses of 297, 429 and 622 μm, respectively, when cutting speed is 10 m/s. Similar to the situation shown in Figure 5, the compressive stress is distributed in the chips on the tool rake face and in the material near the shear zone. The maximum compressive stress on the shear plane is 235 MPa, 240 MPa and 280 MPa, respectively, and the maximum compressive stress at an unformed chip thickness of 10 μm increases by 20% compared with that at the unformed chip thickness of 297 nm.

Figure 9d–f show the KDP crystal temperature distribution with unformed chip thicknesses of 297 nm, 429 nm and 622 nm, respectively, when cutting speed is 10 m/s. The maximum temperatures of the chips are 76 °C, 98 °C, and 117 °C.

In Figure 9, (1) when the cutting speed is 10 m/s, as the unformed chip thickness increases, the cutting force at 10 μm is 140% higher than that at 297 nm; however, the maximum compressive stress increases by only 20%. (2) The maximum chip temperature increases by 28% when the unformed chip thickness is 429 nm compared with 2 μm, while the maximum chip temperature increases by 19% when the unformed chip thickness is 622 nm compared with 429 nm. As the depth of the cut increases, the amount of chips increases, and the amount of chips rubbed against the tool will remain in a relatively stable range to a certain extent, and the frictional heat generation will be stable, so the temperature growth rate will decrease.

### 4.5. Chip Morphology

**Figure 10:**
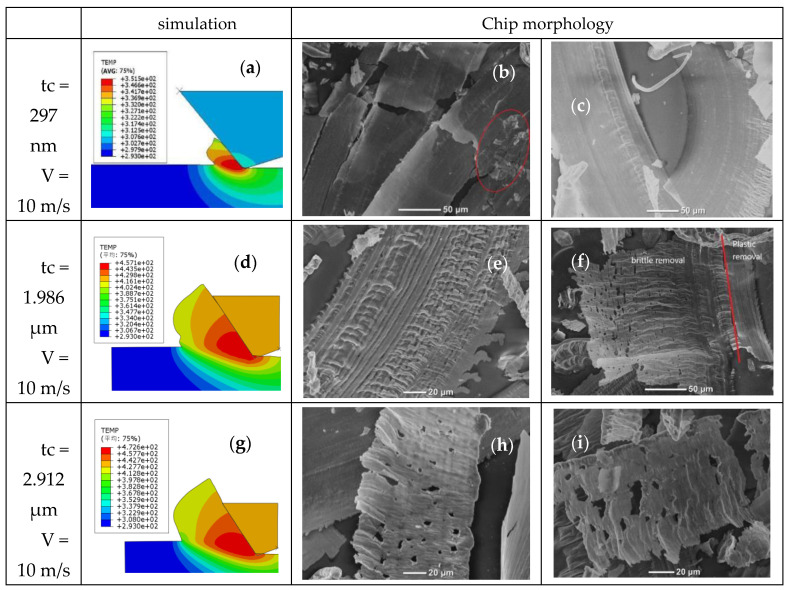
Chip morphology of KDP crystal at different processing stages: (**a**) Temperature of tc = 297 nm, V = 10 m/s; (**b**,**c**) Finishing chip morphology; (**d**) Temperature of tc = 1.986 μm, V = 10 m/s; (**e**,**f**) semi-finished chip morphology; (**g**) Temperature of tc = 2.912 μm, V = 10 m/s; (**h**,**i**) Roughing chip morphology.

Figure 10a shows the KDP chip produced by finishing. The max value of the unformed chip thickness is about 297 nm and the cutting speed is 10 m/s. It shows the cutting temperature is about 70 °C. It is not difficult to find that the chip is continuous and strip-like, and the surface is smooth and complete, so that low temperature has little influence on the KDP crystal structure, and such chip morphology is ideal (Figure 10b,c).

The max value of the unformed chip thickness is about 1.986 μm. From the simulation results in Figure 10d, the cutting temperature is 184 °C. In Figure 10e, we can see that the chip is continuous, but the surface is wrinkled, which means the material is softened and can be more easily deformed, and the chips are produced quickly and accumulated obviously, so the softening chips become wrinkled in the discharging process. In Figure 10f, there is an obvious brittle plastic transformation, and the right side of the chip has a completely smooth appearance because the removal mode of this chip is plastic material removal, while on the left side of the chip, the chip exhibits obvious wrinkles and pore-like morphology because the removal mode is brittle material removal. The main reason for this phenomenon is that the actual undeformed cutting thickness can be increased along the arc-edge tool, which is illustrated in Figure 1b. In one process, the thickness of the two sides of the chip is different. The material on the side whose chip thickness is less than the depth of brittle–plastic transition is removed plastically, and the quality of the chip is better, while the material on the side whose chip thickness is greater than the depth of brittle–plastic transition is mainly removed brittly; therefore, the chip morphology is incomplete and the quality is poor.

The max value of the unformed chip thickness is about 2.912 μm. From the simulation results in Figure 10g, the cutting temperature is about 199 °C. In Figure 10h,i, it can be seen that the chip shows not only a small segment with obvious fractures, but the surface has obvious wrinkles with serious damage, and at the same time, the burrs on the edge of the chip are melted at a high temperature. In the case of high speed and large unformed chip thickness such as rough processing, the chip temperature can be higher than the phase transition temperature, which makes the material easier to deform and phase transition, so the chip is easier to perforate and melt. 

## 5. Discussion

In this paper, the force and heat changes under different working conditions were analyzed by finite element simulation and experimental research, the chips’ morphology under actual working conditions was observed, and then the mechanism of chip formation was analyzed. Next, the structure of KDP crystal can be analyzed and discussed through the existing results and simulations. 

First of all, in the simulation and experimental results, under the cutting parameters we used in the KDP finishing, the temperature can reach only 140 °C, which is less than the dehydration temperature of KDP crystal (about 182.3 °C). However, in the KDP semi-finishing and roughing, the unformed chip thickness is 5~9 times higher than that in finishing, so the temperature can reach 184 °C~199 °C, and KDP crystals are more likely to dehydrate or deform.

Secondly, in the KDP crystal cutting, taking the cutting speed of 15 m/s and the max value of the unformed chip thickness of 429 nm as an example, the chip temperature measurement value reaches 112 °C, and the temperature difference within the crystal easily exceeds 4 °C. In Figure 8e, the max value of the tensile stress is 7.792 MPa, which exceeds the tensile strength limit of 6.67 MPa. In summary, this shows that the value of tensile stress is likely to be bigger than the tensile strength of KDP crystal under the combined action of the stress produced by crystal removal and the thermal stress of temperature, resulting in micro-cracks on the machined surface and sub-surface.

Thirdly, the phenomenon that chip morphology changes obviously in Figure 10e,f,h,i is explained as follows: in the cutting process, the cutting force and temperature act together on the KDP crystal, causing material properties such as hardness and specific heat capacity to change. Then, these changes in material properties will affect the cutting force and cutting temperature to achieve a stable state. In this state, the cutting stress is greater than the compressive strength of the material, and high-temperature chips break. At the same time, the local temperature of the chip has exceeded the phase transition temperature of the KDP crystal, and the material will easily generate a dehydration reaction, and even change the structure and morphology. After the chip is separated from the workpiece, the temperature gradually decreases. The residual stress makes the internal stress of the chip and the processed surface extremely unbalanced, likely causing secondary damage. Thus, microcracks may appear on the surface of the workpiece, and the chips will have perforations and edge melting.

## 6. Conclusions

In this paper, the cutting process of KDP crystal is studied by combining finite element simulations, measurement experiments and morphological observation experiments. The main conclusions of this paper are as follows:

1. In this paper, the KDP crystal fly-cutting simulation model is established, the stress field, cutting force and cutting temperature are calculated, and the comparison experiment is carried out to verify the validity of the model. The maximum difference between the simulation temperature and the experimental results is less than 10 °C, which shows that the accuracy of the finite element simulation model can be accepted.
2. The chip morphology of KDP crystal has been observed. Based on the finite element simulation results of the stress field and temperature field, the causes of chip morphology formation and surface cracks of the workpiece have been analyzed.
3. When the max value of the unformed chip thickness is 429 nm, and cutting speed is 15 m/s, the measured chip temperature reaches approximately 110 °C, and at the time there is also a residual temperature of 30 °C~50 °C on the machined surface and a tensile stress of 7.792 MPa, possibly resulting in micro-cracks on the machined surface and sub-surface.
4. In the case of semi-finishing and roughing, the chip temperature can reach 184 °C or higher, and it is quite possible for the KDP crystal to undergo a phase change reaction in the fly-cutting process, which makes the material easier to deform, and the chip is easier to perforate and melt, while higher residual temperatures can more easily damage the machined surface.

## Figures and Tables

**Table 1:** Natural diamond material properties.

Physical Quantity	Value
Elastic modulus (Gpa))	1114
Poisson ratio	0.07
Linear expansion coefficient (μm/(m⋅°C))	1.18
Specific heat capacity (*J/(Kg °C*))	507.9
Thermal conductivity (*W/(m °C)*)	2000
Density ($kg/m^{3}$)	3520

**Table 2:** Stiffness constant value [22].

*C*_11_	*C*_12_	*C*_13_	*C*_33_	*C*_44_	*C*_66_
71.6 GPa	−6.3 GPa	14.9 GPa	56.4 GPa	12.5 GPa	6.2 GPa

**Table 3:** Thermal parameters of diamond tools and type II KDP crystals.

Parameters	Diamond	KDP Crystal		
*α[μm/(m°C)]*	2000	*α_x_*	*α_y_*	*α_z_*
		16.1	16.1	29.0
*λ(10^−6^/°C)*	1.18	*λ_x_*	*λ_y_*	*λ_z_*
		2.0	2.0	3.0
C*[J/(kg°C)]*	502	$\begin{array}{l} -120+4.56T-7.38(10^{-3}{)T}^{2}+6.59(10^{-6}{)T}^{3}-3.05(10^{-9}{)T}^{4}+5.72(10^{-13})T^{5}\\ (273K<T<1500K) \end{array}$		
*k*	0.03	0.36		

**Table 4:** Diamond tool parameters.

Rake Angle	Flank Angle	Nose Radius	Edge Radius
−45°	18°	5 mm	120 nm

**Table 5:** Experimental design parameters.

Parameters	Value
cutting speed	3 m/s, 5 m/s, 8 m/s, 10 m/s, 15 m/s
feed	10 μm/rev
the max value of the unformed chip thickness (cutting depth)	297 nm (2 μm), 429 nm (5 μm), 622 nm(10 μm)
